# Supporting Diversity in Science through Social Networking

**DOI:** 10.1371/journal.pbio.1001740

**Published:** 2013-12-31

**Authors:** Giovanna Guerrero-Medina, Mónica Feliú-Mójer, Wilson González-Espada, Greetchen Díaz-Muñoz, Marcos López, Samuel L. Díaz-Muñoz, Yaihara Fortis-Santiago, Jacqueline Flores-Otero, David Craig, Daniel A. Colón-Ramos

**Affiliations:** 1 Ciencia Puerto Rico, San Juan, Puerto Rico, United States of America; 2 Program in Neuroscience, Department of Neurobiology, Harvard Medical School, Boston, Massachusetts, United States of America; 3 Department of Mathematics, Computer Science and Physics, Morehead State University, Morehead, Kentucky, United States of America; 4 Nebraska Center for Virology, Morrison Research Center, Lincoln, Nebraska, United States of America; 5 Vilano Research Group, Biotechnology, Innovation and Technology Development, Fundación Cardiovascular de Colombia, Floridablanca, Colombia; 6 Program in Biomedical Sciences, Universidad del Valle, Ciudad Universitaria Melendez, Cali, Valle del Cauca, Colombia; 7 Section of Ecology, Behavior and Evolution, University of California San Diego, La Jolla, California, United States of America; 8 AAAS Science and Technology Policy Fellow, Washington (D.C.), United States of America; 9 Institute of Neurobiology, University of Puerto Rico, San Juan, Puerto Rico, United States of America; 10 Stanford Hospital and Clinics, Stanford, California, United States of America; 11 Program in Cellular Neuroscience, Neurodegeneration and Repair, Department of Cell Biology, Yale University School of Medicine, New Haven, Connecticut, United States of America

## Abstract

In this Community Page, we learn how a scientific community leverages social networking tools to connect a group of dispersed scientific researchers in Ciencia Puerto Rico; this effort fosters innovative research and educational collaborations and changes the way scientists interact with the public.

## Introduction

Science is disproportionately produced at research centers within a few select regions [1],[2]. This distribution contributes to “brain drain”—the cultural and geographical separation of researchers from their communities of origin [3]. In places lacking research centers, brain drain precludes achieving a critical mass of scientific expertise and the development of science, technology, engineering, and mathematics (STEM). Displaced scientists gradually become disconnected from their home communities and colleagues, presenting a challenge to maintaining research collaborations that could benefit their communities of origin. Insidiously, dispersion also presents socio-cognitive challenges to scientists who see themselves as underrepresented in the larger culture of science [4]–[6].

Social networks hold enormous promise for “connecting” dispersed groups and providing new opportunities for fellowship and mentorship among underrepresented communities in science. Faced personally with these obstacles, in 2006, we created Ciencia Puerto Rico (CienciaPR; www.cienciapr.org), an online network that connects scientists with geographic, academic, and/or cultural ties to Puerto Rico. CienciaPR was built to counteract the negative effects of scientific brain drain by: (1) promoting scholarly interaction among self-identified members of an otherwise dispersed community; (2) providing visibility to diverse scientific role models; and (3) supporting research and science education through initiatives that culturally resonate with our community of origin.

Here, we present CienciaPR's design and discuss how we leverage our membership to enhance science education and mentoring of Puerto Rican students. Looking beyond our own community, we suggest how our efforts can be translated to similarly dispersed populations. By growing and supporting scientific diversity, we believe social networking can democratize the scientific enterprise and more broadly distribute its benefits.

## Conceptualization and Implementation of CienciaPR

The Puerto Rican scientific community is highly dispersed—64% of Puerto Rican PhD STEM students and 44% of the Puerto Rican STEM doctorate workforce resides outside the Puerto Rican archipelago [7]–[9]. Recent emigration trends have exacerbated dispersion [10]. A virtual space that connects the Puerto Rican scientific community thus represents a powerful means of addressing the unique challenges faced by this population, which shares elements of both diaspora and minority communities.

Two aspects were key in the design of CienciaPR. First, we conceptualized the network as a site for “anyone interested in science and Puerto Rico.” This definition was broad by design to account for the idiosyncrasies of cultural, ethnic, and national identification, and to promote the inclusion of anyone interested in contributing to the Puerto Rican scientific community regardless of their place of origin. While most of our membership consists of Puerto Rican scientists, a broad definition helps attract scientists around the world interested in research or educational collaborations with Puerto Rico.

**Figure 1:**
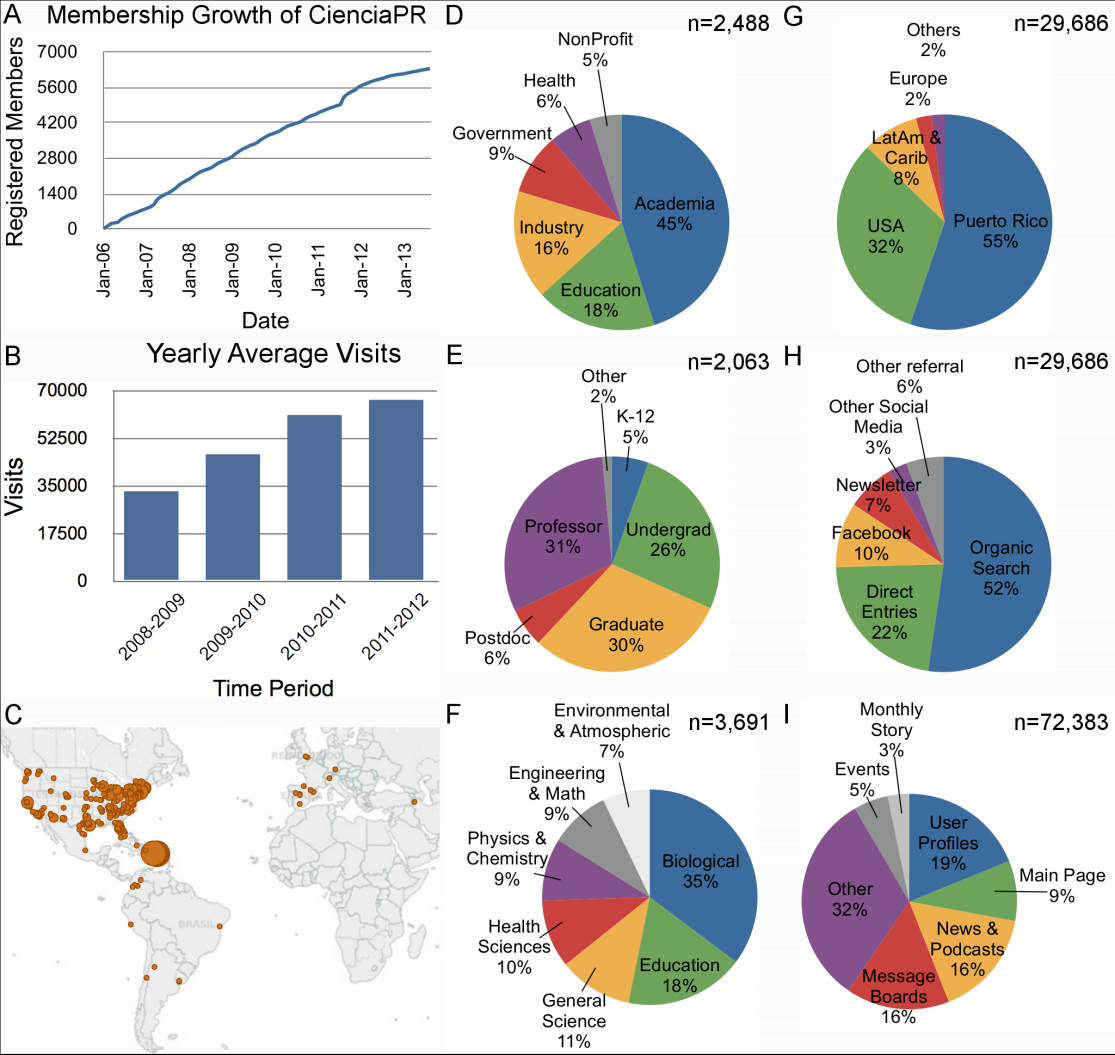
CienciaPR website use and member characteristics. (A) Number of members who have registered with CienciaPR since 2006. (B) Average yearly visits to CienciaPR.org, from October 2008–September 2012, based on Webalizer data. CienciaPR switched to GoogleAnalytics after September 2012. (C) Map representation of CienciaPR members' geographic dispersion (portion of map is cropped for display purposes). CienciaPR members are in 48 countries, 47 states (not including the territory of Puerto Rico), and over 185 universities in the US. Map made with Tableau Public (D–F) Distribution of members based on (D) work sector, (E) training stage, and (F) broad scientific discipline of interest. Not all members indicated this information on their profiles. Percentages are based on total number of respondents (n). Data as of September 8, 2013. (G–I) Recent CienciaPR website activity from June 1, 2013–August 31, 2013 based on Google Analytics for (G) website visits by geographic region, (H) website visits by source, and (I) page views by website section. Percentages are based on (n), total number of visits (G,H) or pageviews (I).

Second, we gave important consideration to user profiles, a typical feature of social networking sites [11], but tailored them for scientists, gathering information about research interests, publications, institutional affiliation, mentoring, entrepreneurial interests, and training history (see http://bit.ly/1bXN6dy, for example). At CienciaPR.org, users can open and populate a profile, free of cost. During the first 6 years of the website, name and email were the only fields required to open a profile. Despite this, a majority of CienciaPR members have chosen to list their institution (54%), field(s) of scientific interest (58%), and training or work position (70%). Information collected through CienciaPR profiles allows the creation of a map of the community's geographic footprint and collective capacity (Figure 1C). Profiles serve both to identify individuals with specific expertise for mentoring or collaboration, and to provide visibility to a community of scientists, otherwise invisible due to geographic dispersion and underrepresentation.

A number of social networking tools (peer-to-peer messaging, maps of nearby members, message boards, events calendar, personal scientific blogs, and automated matching of members with similar interests) facilitate interaction and keep the website dynamic and updated through member-driven content. In addition, we add value by creating listings—many updated in real-time through real simple syndication (RSS)—that display funding opportunities, job openings, and Puerto Rican scientific organizations. This approach allows us to crowd-source relevant content in a cost-effective manner, publishing an average of seven new pieces of member-driven content per week.

The original CienciaPR website, developed pro bono by one of the authors (DC), consisted of an SQL database with a custom-designed front-end and content management system. This proved sufficient for most of our operations during the first 6 years. The community's interest in our website, as evidenced by member registrations and visits (Figure 1A and 1B), served to secure funding from the Puerto Rico Science, Technology, and Research Trust for the development of a more sophisticated Drupal7-based platform (launched September 2012). Both versions of the website were intentionally designed with open source platforms to facilitate sharing and use by other communities. Though CienciaPR chose Drupal for its customizability, there are other simple and affordable social networking solutions (e.g., Ning, SocialEngine, Elgg, BuddyPress) available to groups interested in replicating our approach.

News about the launch of the original website was initially sent to scientific leaders in Puerto Rico but thereafter membership continued to grow with no marketing efforts, based on word-of-mouth and organic website traffic. In 7 years CienciaPR's membership has soared to 6,364 (as of September 8, 2013), making it the largest database of Puerto Rican scientists across the world (Figure 1C–1F). Based on national surveys, we estimate CienciaPR's membership accounts for ∼30% of all Puerto Rican doctorates in STEM and 65%–70% of all Puerto Rican graduate students across the US [12].

CienciaPR has an engaged following. Almost 29% of traffic to the website is from direct sources (Figure 1H; direct URL entries and member newsletter) and CienciaPR's Facebook page has a weekly engagement average of 691 unique users. Member profiles and our message boards—displaying research training and funding opportunities—are our most frequented sections (Figure 1I). Membership engagement is reflected in the thousands of peer-messages and board posts over the lifespan of the website.

## From Connectivity to Action

CienciaPR's social network enables the identification of both needs and resources within our community for innovative research and educational interventions. Below, we enumerate online and offline initiatives, describe how they emerged organically from the needs of our membership, and how online tools were used to leverage the knowledge-base of the network to arrive at crowd-sourced, community-driven solutions.

## Culturally Relevant Science Communication and Education

Science learning is enhanced when concepts are made relevant to an individual's context and culture [13]–[18]. Yet in Puerto Rico, as in most minority communities and developing countries, scientific concepts are seldom illustrated in a culturally relevant fashion [19]–[21].

Scientists can play a key role in explaining the meaning and importance of research findings to their communities [22]–[26]. CienciaPR has leveraged its unique network to enhance formal and informal science education in Puerto Rico and among Spanish-speaking communities through collaborations with eight national and international media outlets (http://bit.ly/1a9xCzy) [27]. We established collaborations by emailing newspaper editors or bloggers with representative articles that could be published in their venue. In our experience, many media outlets lack access to scientific content and most venues we contacted responded eagerly. The main challenge was ensuring scientific topics were appropriately explained for a lay audience.

To address this hurdle, early on we identified two volunteer editors within our network (MFM and WGE) who could serve as key liaisons between CienciaPR members and media partners. Written work submitted by our members is first curated and edited by these expert science communicators before being sent to media collaborators. We prioritize articles that talk about science performed in Puerto Rico or by Puerto Rican scientists or that make universal science concepts meaningful and relatable by using examples from the Puerto Rican context. While partners reserve the right to publish the articles submitted by CienciaPR, 89% have been published (http://bit.ly/1frwIoj). Some articles are also distributed as podcasts (through CienciaPR.org and iTunes) and broadcasted through local radio stations (http://bit.ly/16uTo3h).

In 2006, before CienciaPR's collaboration, very few of the science stories in El Nuevo Día (the main Puerto Rican newspaper) were contributed by scientific experts [19]. CienciaPR's media collaborations have increased the amount of culturally relevant scientific news content in Puerto Rico with 385 articles and 210 podcasts released as of October 2013, all authored by scientists.

 Inspired by its successful media collaborations, in 2009 CienciaPR crowd-sourced a collection of short essays about science for the general public. Twenty-three scientists contributed 61 essays to the book *¡Ciencia Boricua! Ensayos y Anécdotas del Científico Puertorro* [28]. The articles showcase world-class research performed in Puerto Rico, or by Puerto Ricans, and research performed internationally. In all cases, essays illustrate scientific concepts using examples from the Puerto Rican context, culture, and landscape. This approach serves to underscore the relevance of international scientific discoveries locally, as well as celebrate local contributions to the international scientific enterprise.

Through our network we learned that teachers were using the book to help contextualize science in their curricula. Although not developed as a textbook, we followed teachers' lead and, with the book as centerpiece, undertook a pilot project to increase Puerto Rican elementary and middle school students' interest in science. Results from the pilot study suggest that students' interest in science increased after exposure to context-relevant material [29].

## Providing Role Models and Advancing Careers

Young people's perception of scientists and their ability to envision a successful scientific future is influenced by access to diverse representations of scientists [30]–[34]. We have placed a high priority on increasing the visibility of Puerto Rican and Hispanic scientific role models by maintaining member profiles publicly accessible, celebrating their successes through our blog and social media accounts, and increasing their coverage in news media (see above). Additionally, we publish monthly bilingual online features (67 as of October 2013) highlighting the life and work of accomplished members, showcasing their successes, but also their challenges, drive to succeed, and interest in giving back to their communities.

Our efforts extend beyond our website by organizing offline events that promote community and mentoring. For instance, we coordinate annual social events during national scientific conferences (e.g., Society for Neuroscience, Ecological Society of America, American Society for Microbiology) that give attendees the opportunity to network and thus mitigate the effects of dispersion. These events are often organized in collaboration with Puerto Rican chapters or members of the scientific societies.

CienciaPR has also organized meetings to tackle issues such as the quality of science education in Puerto Rico and career advice to scientists-in-training. Connections with academic leaders and institutions in Puerto Rico have been crucial for these endeavors. For example, in 2011, in collaboration with the Research Centers in Minority Institutions (NIH-RCMI) of the University of Puerto Rico, CienciaPR held a symposium designed to guide students interested in STEM careers, but who lacked access to research-intensive environments. Over 120 students from 15 college campuses across Puerto Rico attended presentations that discussed how to apply, be successful, and find funding towards the completion of doctorate degrees in biomedical research. Eighty-eight percent of students felt the symposium “helped clarify their interest in a graduate science program” and 90% indicated that “they were interested in pursuing a scientific career.”

## Challenges and Lessons Learned

Though our network is at the heart of CienciaPR, it would not have reached its full potential without a team of volunteers who emerged as CienciaPR's governing and operating structure (Box 1). We have found two main challenges in running a grassroots, non-profit organization led by volunteers: limited funds and limited availability of human resources. Community members are eager to make time to volunteer, because they appreciate the potential of CienciaPR to help their careers and give back to their community. Nonetheless, academic and professional careers force time and effort commitments to be variable. Though most of CienciaPR's initiatives have been implemented with minimal funding, in-kind contributions, and through the donation of volunteers' time, much more could be achieved with additional funds and full-time staff. Lack of these resources has restricted the potential reach and impact of the organization and resulted in lost opportunities. Though the use of social networking tools in combination with community-based volunteer leadership has resulted in the initiation of much needed interventions, we are currently working to establish more structured governance and a sustainable financial plan to build on the success of our initial years.

**Box 1. CienciaPR's Volunteer Leadership at Work**

CienciaPR has established a volunteer-based governing and operating structure (http://bit.ly/18JLYHi). To aid others interested in this approach we answer key questions regarding our leadership model:

**How many volunteers are needed to run CienciaPR?** The current CienciaPR Team consists of nine PhD scientists. Additionally, seven volunteers help with the online platform and other initiatives.

**How do you find volunteers?** Volunteers self-identify through our network (through email or by completing a webform). After discussing their interests, time, and skills, they are assigned to functions or projects as needed. Over the years, a group of leaders has emerged through their dedication and contributions. There is no lack of volunteers (we have a waitlist of 52), and in fact one challenge is the ability to manage the number of people interested in helping out.

**How do volunteers find time?** It is challenging to find time amidst the demands of a scientific career, but we have found that volunteers are motivated by their desire to give back to the community. We avoid overextending our volunteers by discussing their time availability and matching their tasks with their career goals and interests.

**How is the online platform managed?** The CienciaPR website has an easy-to-use content management system that keeps operational costs down. One of our team members (GGM) manages the website, develops new website sections, and performs basic web maintenance. She trains and supervises other volunteers who contribute curated content (e.g., news, events, training and funding opportunities).

**How are initiatives created and implemented?** CienciaPR's projects are conceived and implemented by members and volunteers. This contrasts markedly from conventional organizations, where staff devises initiatives without much participation from its audience. This crowd-sourced, community-driven approach means initiatives are directly responsive to perceived needs, breeds creativity, and assures that volunteers are matched to efforts that interest them. The participatory nature of volunteer leadership has driven the enhancement of the network and future projects.

**How is the sense of community maintained among leadership?** Though the CienciaPR Team is as geographically dispersed as the CienciaPR network, volunteers are in almost daily contact via email, and hold monthly teleconferences where we discuss projects, roles, and assignments. CienciaPR's open and participatory decision-making process enables volunteers to contribute ideas and voice concerns. The team's bond is further solidified through regular celebrations and recognitions of volunteers' contributions and achievements (within or outside of CienciaPR).

## Replicability to Other Communities

Networks of diaspora or minority scientists are uniquely positioned to contribute to their communities by counteracting geographic dispersion and isolation [35]–[41]. CienciaPR has demonstrated that an online approach can be leveraged to mitigate these dual challenges. We believe certain elements have enabled the growth and impact of CienciaPR: a social network approach, community-sourced initiatives, and collaboration with local scientists. While our efforts have focused on Puerto Rico, these aspects can enable other dispersed groups to connect with home communities for outreach, education, research collaboration, or entrepreneurial efforts. Organizations such as the Society for the Advancement of Hispanics/Chicanos and Native Americans in Science (SACNAS) (sacnas.org), Just Garcia Hill (justgarciahill.org), Minority Postdoc (minoritypostdoc.org), and Scientista Foundation (scientistafoundation.com), to mention a few, have also demonstrated the power of online networks to address the challenges facing minorities in science. With CienciaPR, we took this concept and extended it beyond mentoring and career advice, to connect scientists with their community for educational outreach and science communication.

Broadening the participation of underrepresented groups across the world of science is critical for the progress of research and the development of countries [42],[43]. CienciaPR stands as an example of a social networking model applied to geographically dispersed scientists that is cost-effective, feasible, and impactful. We extend our hand to other groups that want to use online technologies to apply their passion for science and desire to give back to their communities.
